# High rates of adherence and treatment success in a public and public-private HIV clinic in India: potential benefits of standardized national care delivery systems

**DOI:** 10.1186/1472-6963-11-277

**Published:** 2011-10-17

**Authors:** Anita Shet, Ayesha DeCosta, Elsa Heylen, Suresh Shastri, Sara Chandy, Maria Ekstrand

**Affiliations:** 1 Department of Pediatrics, St John's National Academy of Health Sciences, Bangalore, India; 2 Division of International Health, Karolinska Institutet, Stockholm, Sweden; 3 Research Institute, St John's National Academy of Health Sciences, Bangalore, India; 4 Department of Medicine, University of California, San Francisco, USA; 5 National AIDS Control Organization, India; 6 Department of Medicine, John's National Academy of Health Sciences, Bangalore, India

## Abstract

**Background:**

The massive scale-up of antiretroviral treatment (ART) access worldwide has brought tremendous benefit to populations affected by HIV/AIDS. Optimising HIV care in countries with diverse medical systems is critical; however data on best practices for HIV healthcare delivery in resource-constrained settings are limited. This study aimed to understand patient characteristics and treatment outcomes from different HIV healthcare settings in Bangalore, India.

**Methods:**

Participants from public, private and public-private HIV healthcare settings were recruited between 2007 and 2009 and were administered structured interviews by trained staff. Self-reported adherence was measured using the visual analogue scale to capture adherence over the past month, and a history of treatment interruptions (defined as having missed medications for more than 48 hours in the past three months). In addition, CD4 count and viral load (VL) were measured; genotyping for drug resistance-associated mutations was performed on those who were in virological failure (VL > 1000 copies/ml).

**Results:**

A total of 471 individuals were included in the analysis (263 from the public facility, 149 from the public-private facility and 59 from the private center). Private facility patients were more likely to be male, with higher education levels and incomes. More participants reported ≥ 95% adherence among public and public-private groups compared to private participants (public 97%; private 88%; public-private 93%, p < 0.05). Treatment interruptions were lowest among public participants (1%, 10%, 5% respectively, p < 0.001). Although longer clinic waiting times were experienced by more public participants (48%, compared to private 27%, public-private 19%, p < 0.001), adherence barriers were highest among private (31%) compared with public (10%) and public-private (17%, p < 0.001) participants. Viral load was detectable in 13% public, 22% private and 9% public-private participants (p < 0.05) suggesting fewer treatment failures among public and public-private settings. Drug resistance mutations were found more frequently among private facility patients (20%) compared to those from the public (9%) or public-private facility (8%, p < 0.05).

**Conclusions:**

Adherence and treatment success was significantly higher among patients from public and public-private settings compared with patients from private facilities. These results suggest a possible benefit of the standardized care delivery system established in public and public-private health facilities where counselling by a multi-disciplinary team of workers is integral to provision of ART. Strengthening and increasing public-private partnerships can enhance the success of national ART programs.

## Background

Although the HIV epidemic may show signs of levelling off in some parts of the world [1], the overwhelmingly high numbers of people living with HIV in Asia and Sub-Saharan Africa continue to fuel efforts in capacity-building, improving existing infrastructure and widening antiretroviral therapy (ART) access in areas that are most affected. The United Nations Secretary-General, Mr. Kofi Annan, stated in 2001 that,*"People no longer accept that the sick and dying, simply because they are poor, should be denied drugs which have transformed the lives of others who are better off" *[2]. It was with Kofi Annan's lofty goal in mind that in 2004, the political leaders in India and the National AIDS Control Organization (NACO) initiated the National AIDS Control Program that provided free antiretroviral treatment access to all those in the nation who had a medical need for these drugs. Starting with just eight centers in 2004, this Program grew exponentially, and currently there are almost 300 ART Centers across the country serving over 360,000 adults and 22,000 children with antiretroviral medications [3]. Today, much of the HIV care, treatment and support in India is provided through NACO-run ART centres [3]. Following a diagnosis of HIV infection and assessment of the need for medication, patients within these ART Centres are provided first-line ART free of cost along with intensive counselling at each visit. Services in these centers are fully financed and delivered by the public sector in accordance with strict treatment guidelines set by the National Program [4].

Although the bulk of the HIV healthcare system is handled by the national government, it is important to note that the health sector in India is highly pluralistic; multiple systems of medicine including alternative and indigenous medicine are legally practiced in diverse institutional settings [5]. The country also has one the most highly privatized health care systems in the world; out-of-pocket payments account for 72% of total healthcare spending in India [6]. HIV care is also delivered in the private sector where approximately 35,000-50,000 patients are managed by a private physician of their choice, and payment for consultation and treatment are largely made out-of-pocket [7,8]. While there also exists a small philanthropic private sector that includes non-governmental organizations that medically and financially support disadvantaged patients, this paper refers to the formal private health care sector where patients pay for their treatment. With the aim of increasing access to ART, the National AIDS Control Program in India has partnered with reputed private institutions to deliver ART through an innovative partnership approach. The public-private partnership here is a collaboration between the government National AIDS control program and the private entity, wherein the former provides first-line ART free of cost to all patients registered at the center, while the private hospital provides the premises, infrastructure and human resources. There are fifteen such public-private partnership ART Centers in the country at present, which altogether provide care to over 21,000 patients [3]. The public-private centers also operate under the standardized national program treatment guidelines.

As there is wide variation in patient demographics, motives for attendance at a particular sector, system of medical care and drug dispensation methods among these three healthcare systems, it is likely that adherence levels and treatment outcomes also vary among the three settings. In particular, we hypothesized that the standardized system consisting of a high emphasis on counselling and multidisciplinary approach present within the public HIV healthcare system, will have a positive impact on adherence levels among patients and hence treatment outcome. The objective of this descriptive paper was to compare the differences in patient characteristics, adherence, indicators of care, virological failure and drug resistance among fully public, public-private and fully private HIV care-giving centers in Bangalore, India.

## Methods

### Study setting

This study was done at three types of clinics in South India: public, public-private, and fully private. (i) The public facility was represented by government ART Centers located at large state-run public hospitals, predominantly the Bowring and Lady Curzon Hospital. The public centers operate under the NACO program guidelines. Briefly, the NACO program insists on multi-disciplinary care with comprehensive services provided by a physician, nurse, counsellor, and pharmacist within the same setting for each patient, with great emphasis placed on intense counselling sessions prior to initiation of ART and at each subsequent visit. (ii) The public-private facility was the ART Center at St. John's Hospital, a missionary teaching hospital. The public-private partnership here is a collaboration between NACO and the teaching hospital, wherein the former provides NACO-recommended fixed dose combination first-line ART to all patients registered at the center, while the private teaching hospital provides the premises, infrastructure and human resources. The public-private center also operates under the NACO program guidelines. All patients attending the first two types of clinics, the public and the public-private facility, are treated free of cost at the point of service. (iii) The private facility was the out-patient department at St. John's Hospital, where patients are treated independently by their respective physicians and out-of-pocket payments for health services and medications are made by the patients themselves.

### Study populations and design

Study participants included HIV-infected adults taking regular ART who were part of a two-year observational cohort to study adherence at the three centers [9]. Potential participants were first referred by their physicians or counsellors, and those who agreed were escorted to a separate room for informed consent and an approximately 1-hour study interview. The recruitment procedure remained uniform across all the health care settings. Patients in the public and private clinics were recruited over a two year period beginning in August 2007. Patients in the public-private clinic were recruited from March 2008 after the inception of the ART Center. Attendance of the patients was exclusive to a particular clinic, and only those patients who had been with each clinic for a minimum consecutive period of six months were included in this analysis. Cross-sectional data from these patients at six months after recruitment into the study were used in this analysis.

### Data collection

Participants were referred to the study by their physicians, by the outpatient clinic clerk, or by screening staff at outpatient clinic registration. Following administration of informed consent, a face-to-face interview was administered by trained study staff members whoS were not part of the regular clinical care-giving team. The interview questionnaire was developed in English, translated into Kannada, Tamil and Telugu, and independently back-translated into English to ensure semantic equivalence [10]. The interview lasted approximately one hour and assessed a variety of topics, including demographics and health history, medical regimen, adherence barriers and other psychosocial factors. Health history included time of first diagnosis and initiation of ART as well as number and reason for health care visits in the past 3 months. Information was recorded on perceived adherence and psychosocial barriers such as (i) lack of routine barriers (forgetting, being away from home, being busy with other events) (ii) refill related barriers (didn't want to purchase in same community, ran out before refill possible, no money, no stock) (iii) regimen-related barriers (side effects, feeling that medicines were harmful) and (iv) health-related barriers (feeling sick or depressed, feeling healthy enough to skip, alcohol abuse). Disclosure and perceived adherence support were assessed for different categories of people such as spouse, parents, children, siblings, other relatives, friends or co-workers. Patients also were asked about their ease of communication with their doctor, and potential barriers to keeping appointments such as long clinic waiting times or lack of child care.

Adherence measurement: Self reported adherence for the last one month was measured using the visual analogue scale (VAS), which has been validated as a simple and useful measure of self-reported adherence [11,12]. The VAS is a horizontal line ranging from 0 to 100, on which respondents point to a spot on the line that corresponds to the percentage of prescribed pills taken. Treatment interruptions were defined as having missed medications for more than 48 hours in the past three months.

Laboratory tests: Blood samples were drawn every six months for measuring CD4 counts (single platform flow cytometry assay; Guava Technologies Inc., Hayward, CA, USA) and plasma viral load (in-house Real Time PCR assay; Molecular Diagnostics and Genetics, Reliance Life Sciences, Mumbai, India). Plasma viral load of < 100 copies/ml was defined as undetectable viral load, consistent with treatment success. Those patients whose viral load at either recruitment or month 6 exceeded 1000 copies/ml were sampled for drug resistance genotyping assays (YRGcare, Chennai, India, using an in-house real time PCR method [13]) to assess the accumulation of mutations over time.

### Statistical analysis

Univariate descriptive statistics were calculated for continuous variables; frequencies and percentages were used for categorical variables, both for the overall sample and according to type of center. Significance of association with type of center was assessed via Kruskal-Wallis tests for continuous variables. For categorical variables, chi-square tests were used to assess overall significance, and its adjusted standardized residuals to assess deviation from chance for each type of center. In addition, logistic regression with robust standard errors was performed for the outcomes related to virological failure and drug resistance. We considered gender, marital status, education, residence), disclosure, adherence barriers, regimen switch, side effects, income and duration of ART (in months) as potential confounders, but, due to limited statistical power, only included those variables that were significantly bivariately related to the outcome, in the multivariate models. Regression analyses were performed in Stata, version 11, while all other analyses were performed in SPSS, version 18.

The study protocol was approved by the Institutional Review Boards at the University of California, San Francisco and the St John's National Academy of Health Sciences, Bangalore.

## Results

### Socio-demographic characteristics

A total of 471 patients met inclusion criteria for this analysis and were studied in the three types of facilities; 263 participants from the public facilities, 59 from the private facility, and 149 from the public-private facility (Table 1). Two-thirds of the patients were male, with a significantly higher proportion of males attending a private facility (p = 0.001). Private facility patients were also more likely to have a higher level of education (p < 0.01) and have higher incomes (p < 0.01). While the public facility had three-quarters of patients residing within the city and another 23% visiting from other parts of the state, the private and public-private facilities received a larger proportion of patients (19% and 18%, respectively, p < 0.001) from neighbouring states. HIV clinical stage before initiation of ART and mean baseline CD4 did not differ significantly among the three groups.

**Table 1:** Socio-demographic characteristics of patients from different clinic settings, n (%)

	*All (n = 471) n (%)*	Type of HIV healthcare setting			
			
		*Public (n = 263)*	*Private (n = 59)*	*Public-private (n = 149)*	p-value
**Gender: Male**	317 (67)	185 (70)	47 (80)	83 (56)	< 0.001

**Age in years: mean (SD)**	37.3 (8.6)	36.9 (8.5)	40.4 (9.6)	36.9 (8.4)	0.013

**Residence**:					< 0.001
	
***Bangalore/Karnataka***	427 (91)	257 (98)	48 (81)	122 (82)	
	
***Neighbour states***	44 (9)	6 (2)	11 (19)	27 (18)	

**Marital status**:					0.700
	
***Married***	332 (70)	182 (69)	44 (75)	106 (71)	
	
***Never married/widowed/separated/divorced***	139 (30)	81 (30)	15 (25)	43 (29)	

**Education:**					0.001
***≥ 10 yrs schooling***	127 (27)	68 (26)	23 (39)	36 (24)	

**Employed**	345 (73)	208 (79)	44 (75)	93 (62)	< 0.001

**Annual income (USD), median (range)**	803 (0-16,056)	803 (0-6,684)	1068 (0-16,056)	535 (0-5,083)	0.001

The median duration of ART for all patients was 22 months (range: 6-54 months; 27 months, 28 months and 11 months for public, private and public-private settings respectively, p < 0.001). Ninety-eight percent of patients in all the centers were on treatment with the standard ART regimen recommended by the national guidelines [4]; 2 nucleoside reverse transcriptase inhibitors (NRTI) plus 1 non-nucleoside reverse transcriptase inhibitors (NNRTI) (100% public, 92% private, and 98% public-private facility patients; p < 0.001). The regimens most commonly included zidovudine or stavudine plus lamivudine as the NRTI backbone and nevirapine or efavirenz as the NNRTI component. Nine of the 10 private sector patients who were not on these regimens were on a tenofovir-containing regime.

### Disclosure of infection status and adherence support

Patients attending a private facility had disclosed their HIV status to a mean of 3.9 categories of persons, compared to 3.5 in the public setting, and 2.5 reported by patients attending a public-private facility (p < 0.001). The spouse was the most common member who was privy to the participants' HIV status. Among married private center participants, 84% had disclosed to their spouse, compared to 66% public and 51% public-private participants (p < 0.001). However the perceived support for adherence that participants received from family members and friends appeared similar across all three categories of participants, as around 80% in each group reported that they received help in taking their medications. Non-relatives had a bigger role in providing adherence support to public facility participants (6%), compared to private (2%) and public-private participants (1%, p < 0.05).

### Health system access

All patients attending public or public-private facilities reported at least one visit to a health care provider within the preceding 3 months. The seven patients who reported no contact with their health care provider within the same period were all attending a private facility. While 91% of patients from the public and the public-private facilities reported a prescription refill as at least part of the reason for their visit, only 58% of private sector patients contacted their healthcare provider for the same reason (p < 0.001). When queried about their perceived ease of communicating with their treating physician, three-quarters of patients (75%) in the public-private facility said they asked their physician questions at every visit, in contrast to 68% and 59% in the private and public facility respectively (p < 0.01). More public clinic patients (48%) perceived difficulty with long clinic waiting times compared to private (27%) and public-private setting patients (19%, p < 0.001).

### Adherence and barriers of adherence

The percent of participants with ≥95% adherence in the past month was 97% in the public setting, 88% in the private facility and 93% in the public-private setting (p < 0.05) (Table 2). Treatment interruptions lasting longer than 48 hours within the past 3 months were reported with greater frequency among those in the private facility (10%) compared to those in the public facility (1%), or the public-private facility (5%) (p = 0.001). A total of 14% (n = 68) of patients across the three facilities reported experiencing at least one adherence barrier. The most common barriers expressed were related to lack of routine (such as being away from home, being busy with other things, and simply forgetting). A quarter of all private clinic patients experienced these "lack of routine" barriers compared with only 11% in the public-private and 8% in the public facilities respectively (p = 0.001). Refill-related barriers were expressed by only 1% of patients, almost exclusively by private facility patients, the major constraint being the ability to pay for the drugs. Drug supply appeared to be consistent and none of the participants reported non-availability of medicines as a barrier.

**Table 2:** Adherence and treatment failure among the three types of healthcare settings: n (%)

	Type of healthcare setting			
	***Public (n = 263)***	***Private (n = 59)***	***Public-private (n = 149)***	***P value***

≥95% adherence^a ^reported in previous one month	256 (97.3)	52 (88.1)	139 (93.3)	0.01

Treatment interruption reported in previous 3 months	2 (0.8)	6 (10.2)	8 (5.4)	0.001

				

Detectable^b ^viral load	35 (13.3)	13 (22.0)	14 (9.4)	0.05

CD4 ≥ 200 cells/mm^3^	207 (78.7)	47 (79.7)	119 (79.9)	n.s.^d^

				

Genotyping^c ^performed	29 (11.0)	15 (25.4)	19 (12.8)	0.019

Drug resistance-associated mutations present	23 (8.7)	12 (20.3)	12 (8.1)	0.029

^a ^Adherence as measured by Visual Analogue Scale (VAS)

^b ^Detectable viral load: > 100 copies/ml

^c ^Genotyping was performed on those with viral load > 1000 copies/ml

^d ^Not significant

### Treatment failure and drug resistance

A detectable viral load was found in 13% of public facility, 22% of private facility, and 9% of public-private patients (p < 0.05) (Table 2). The presence of drug resistance-associated mutations was assessed for those patients who had viral load > 1000 copies/ml (n = 63). More patients from the private center compared to the other centers had their samples sent for genotyping (public 11%; private, 25%; and public-private, 12%; p < 0.05) and drug resistance mutations were present among 20% of the private sector patients compared to less than 10% of patients in the public and public-private setting (p < 0.05) (Table 2). Logistic regression showed that patients from the private facility were 2.7 times more likely to have drug resistance mutations compared to those from the public facility (Unadjusted OR 2.66, 95% CI 1.24-5.73) (Table 3). The increased odds for development for drug resistance among the private facility patients remained significant even after adjusting for duration of antiretroviral therapy, marital status and adherence barriers, which were significant predictors in the bivariate analyses.

**Table 3:** Risk for drug resistance among different clinic settings

Type of healthcare setting	Unadjusted risk for drug resistance			**Adjusted risk adjusted for marital status, barriers of adherence, time on ART **^**a**^		
	***OR***	***95% CI***	***p-value***	***OR***	***95% CI***	***p-value***

Private	2.66	1.24-5.73	0.012	2.53	1.13-5.66	0.02

Public-private	0.91	0.44-1.90	0.809	1.17	0.51-2.69	0.72

Public	1 (reference category)			1 (reference category)		

^**a **^Factors that were not significantly associated were gender, residence, education, income, disclosure status, side effects, and regimen switch.

## Discussion

This detailed comparison of the characteristics and behaviours of patients in different healthcare systems in HIV care in India gives us new insights into healthcare outcomes in relation to healthcare settings. We have found that despite having lower income and higher healthcare barriers such as prolonged clinic waiting times, patients in the public health care facility had significantly better adherence levels, higher viral suppression rates and lower drug resistance prevalence compared to patients accessing care in the private facility, suggesting superior treatment outcomes among patients in the public healthcare setting.

Previous analysis of the entire cohort of 552 participants at baseline demonstrated a strong association between suboptimal adherence, treatment failure and drug resistance [9]. Adherence to ART is influenced by numerous factors, including state of health, travel or migration, adverse effects, stigmatization and financial constraints. Cost of ART has been identified as a barrier of adherence in several studies; in a study from Botswana, adherence was predicted to increase by 20% if cost were removed as a barrier [14]. Among 150 subjects recruited in Tanzania poor adherence and virological failure appeared to be strongly correlated to self-funded ART [15]. Cost of ART was found to be a significant correlate of self-reported adherence among patients attending private clinics in Mumbai [16], and was an important barrier of adherence discussed among a large majority of patients interviewed in Chennai [17]. In the ART in Lower Income Countries study (ART-LINC), a strong relationship was found between receipt of ART and survival, with the greatest survival benefit being when ART was administered free of cost to patients in resource-limited areas [18]. It is conceivable that the removal of cost as an adherence barrier among patients in the public and public-private healthcare systems has a strong role in the adherence and treatment outcomes seen in our study.

The value of an educational and counselling intervention in improving adherence has been well recognized [19]. A Cochrane review concluded that patient support and educational interventions particularly those targeting coping skills, that are administered over the ART initiation period were indeed associated with improved adherence outcomes [20]. Thus the system practised in the public and public-private settings where counselling is an integral part of HIV care is likely to have influenced the higher adherence rates noted in these settings. Although medical providers in private settings do offer adherence support, the lack of a standard counselling protocol often results in a wide range in the quality and impact of counselling support in private clinics.

The past decade has witnessed a massive scale-up of antiretroviral programs in Asia, particularly India. Today, there are 2.3 million persons in India living with HIV, equivalent to approximately 0.3 percent of the adult population, which is a 67% reduction from early prevalence reports in 2005-2006 [3]. India is a large, diverse country with complex social issues which is a challenge to any national medical program. However the country has a well- articulated national strategy which is described in the National AIDS Control Program III (NACP III) [21] and is anchored in the principles of health promotion efforts while seeking to integrate prevention with care, support and treatment. The Program hence utilizes public, nongovernmental and private health institutions to carry out its functions of prevention, care and treatment. The supply chain management of anti-retroviral drugs is managed through a separate team that is responsible for maintaining a continuous supply of ARV drugs. Clearly articulated operational guidelines for ART centers lay the framework for the standard of care that is provided at these centers [22]. Counseling for the patient is emphasized at every stage; from the initial health seeking visit, to diagnosis and evaluation of clinical stage, to initiation and maintenance on ART. ART centers are also linked to community care centres which are set up within the non-governmental organization (NGO) sector with the main objective of providing psycho-social support, ensuring drug adherence and providing home-based care [23]. National data indicate that among all patients who have been initiated on ART so far, 11% have died, 0.65% have discontinued treatment, and only 9% patients are missing or lost-to-followup [23]. Similar results were seen in a systematic study conducted at 3 government ART centers in India [24].

In order to widen their reach towards people from different socio-economic backgrounds, NACO has established several public-private partnerships in NGOs and private medical colleges structured such that both the government and the private institution have an equal role in maintaining the quality of care offered at these centres. It is increasingly believed that under such partnerships, public and private sectors can play innovative roles in financing and providing health care services, particularly in India [25-27]. In the public-private ART center model, the entire systems of counselling, encouragement of treatment supporters during the initial visits during the start of ART, multidisciplinary teamwork and patient flow, are maintained in a similar manner comparable to the system established within the public centers. Our data clearly show that health outcome as measured by adherence levels and virological suppression was clearly optimal in the public-private facility compared to the fully private setting.

Cost of antiretroviral drugs has been implicated as a major factor influencing adherence, although the literature is divided on this issue [28]. An Indian study of HIV-infected patients from Pune and Delhi has shown that adherence was higher among patients paying out-of-pocket compared to those who were receiving free ART via an employee-insurance program; the investigators concluded that provision of free ART without adequate counselling and adherence support was likely to weaken treatment success [29]. Our study also underscores the critical role played by the standardized national system of ensuring counselling and treatment support that is in-built into the ART clinics across the country.

There are several limitations with this study. The numbers studied were relatively small, and the patient numbers were dissimilar in the different settings. The public-private facility was newly begun and was reflected in the short duration of ART that the participants from this setting experienced. The relative youth of the center may have unduly influenced the lower proportion of those with detectable viral load although factors such as systematic counselling and better adherence may have also influenced the outcome. In addition, the private and public-private settings were located within the same institution, although it is important to note here that there was no overlap of patients or medical care providers, and the two settings were geographically separate in different locations. In contrast, the presence of both settings within the same institution may be seen as an advantage; the single geographical location may have minimized the heterogeneity of patients and lent more significance to the positive outcome noted in the public-private facility. It is also possible that these results may be indicative of the individual center and not the public-private or private category on the whole as only one such center each was included in the study. However the relative homogeneity of the organizational capacity of the public centers is assured by the National AIDS Control Program guidelines [21,22], and it is likely that the public-private and private centers included in the study are also representative of other similar settings in the country. Additional research would be necessary to see if these findings can be replicated in other settings. It is noteworthy that since this is a cross-sectional study, no conclusions can be drawn regarding cause and effect. These limitations notwithstanding, the results of this study are strongly suggestive of the larger benefit of including strict standardized counselling guidelines and establishing a multi-faceted paradigm of medical care that is prevalent in the public and public-private HIV care facilities established under the National AIDS Control Program in India.

## Conclusions

The results of this comparison study indicate that, compared to fully private HIV healthcare systems where patients report better employment rates and higher socioeconomic standards, adherence and virological suppression is better maintained within the public and public-private health care setting where counselling is integral to provision of care and ART, and where a multi-disciplinary team of workers are involved in HIV healthcare delivery. While better outcomes are clearly seen in the public and public-private health care settings included in this study, larger multicentric studies are required to support this preliminary information. Overall the results clearly imply that strengthening patient support mechanisms and increasing public-private participation may be an effective global approach towards diminishing the burden of HIV infection worldwide.

## Competing interests

The authors declare that they have no competing interests.

## Authors' contributions

AS conceived the plan to analyse the data according to different health systems. AS and AD wrote the first draft of the manuscript. ME, and SC, conceived the design of the original study, developed the study instruments and implemented the study. EH performed the statistical analyses and created the tables. SS provided valuable feedback on the activities of the National AIDS Control Organization of India. All authors participated in the data interpretation, and have read and approved the final manuscript.

## Pre-publication history

The pre-publication history for this paper can be accessed here:

http://www.biomedcentral.com/1472-6963/11/277/prepub
